# Species and embryo genome origin affect lipid droplets in preimplantation embryos

**DOI:** 10.3389/fcell.2023.1187832

**Published:** 2023-05-12

**Authors:** Paulina Lipinska, Piotr Pawlak, Ewelina Warzych

**Affiliations:** Department of Genetics and Animal Breeding, Poznan University of Life Sciences, Poznan, Poland

**Keywords:** embryo, lipid droplet (LD), lipid metabolism, porcine, bovine, parthenotes

## Abstract

Mammalian embryo development is affected by multiple metabolism processes, among which energy metabolism seems to be crucial. Therefore the ability and the scale of lipids storage in different preimplantation stages might affect embryos quality. The aim of the present studies was to show a complex characterization of lipid droplets (LD) during subsequent embryo developmental stages. It was performed on two species (bovine and porcine) as well as on embryos with different embryo origin [after *in vitro* fertilization (IVF) and after parthenogenetic activation (PA)]. Embryos after IVF/PA were collected at precise time points of development at the following stages: zygote, 2-cell, 4-cell, 8/16-cell, morula, early blastocyst, expanded blastocyst. LD were stained with BODIPY 493/503 dye, embryos were visualized under a confocal microscope and images were analyzed with the ImageJ Fiji software. The following parameters were analyzed: lipid content, LD number, LD size and LD area within the total embryo. The most important results show that lipid parameters in the IVF vs. PA bovine embryos differ at the most crucial moments of embryonic development (zygote, 8–16-cell, blastocyst), indicating possible dysregulations of lipid metabolism in PA embryos. When bovine vs. porcine species are compared, we observe higher lipid content around EGA stage and lower lipid content at the blastocyst stage for bovine embryos, which indicates different demand for energy depending on the species. We conclude that lipid droplets parameters significantly differ among developmental stages and between species but also can be affected by the genome origin.

## 1 Introduction

Mammalian preimplantation embryo development is a complex, well-organized process involving many changes both on a molecular and cellular level. The crucial processes determining embryo quality are, e.g., proper fertilization, the timing of the first cleavage, genome activation and epigenetic reprogramming involving methylation/demethylation and histone modifications (Deutsch et al., 2014; Sudano et al., 2016). Embryo quality may be effectively evaluated in many aspects related to metabolic pathways, including energy metabolism.

The main energy sources for embryos are carbohydrates and lipids (Bradley et al., 2016). Embryos at the early stages of development utilize mainly pyruvate and lactate, while from the morula stage, the glucose requirement increases (Leese, 2012) The energy metabolism within the first cleavages of bovine embryos, up to the embryonic genome activation (EGA) at the 8/16-cell stage, is relatively slow and depends on maternal mRNA, proteins and metabolites collected in the cytoplasm during oocyte growth and maturation (de Lima et al., 2018). Shortly before and following the EGA, there is a noticeable increase in energy demand due to further cell proliferation and differentiation, formation of blastocoel and hatching (Milazzotto et al., 2020; Milazzotto et al., 2022) Energy demand within the blastocysts varies between inner cell mass (ICM) and trophectoderm (TE), since in mice glucose metabolism inhibition negatively affects TE development while ICM remains unchanged (Chi et al., 2020) Embryos can also use amino acids metabolic pathways for energy production, yet studies show that when the amino acid metabolism increases—the bovine embryo quality decreases (Leese, 2012).

An important element of the energy metabolism of oocytes and embryos is the uptake and storage of lipids. Embryos lipidome is sensitive to external factors, e.g., *in vivo* versus *in vitro* system of embryos culture, where higher lipid accumulation is observed in the *in vitro*-produced embryos or in response to stress (Sudano et al., 2011; Janati Idrissi et al., 2021; Melo-Sterza and Poehland, 2021). Moreover, lipid metabolic pathways show distinct activities when subsequent stages of embryo development are compared depending on the species (cattle vs. mouse) or origin (*in vivo* vs. *in vitro*) (Milazzotto et al., 2022). Therefore, lipid metabolism characteristics might be related to embryo quality and considered an important embryo viability marker/parameter (Ghanem et al., 2014; Milazzotto et al., 2016).

Lipids are stored in the cells as triacylglycerols (TAGs) and sterol esters in organelles called lipid droplets (LD). Fatty acids, which are the products of TAGs hydrolysis, might be transferred from LD into mitochondria and metabolized via β-oxidation. Lipid droplets are highly dynamic organelles, able to change their size, number, and location within the cytoplasm. The morphology of lipid droplets may change during embryo development in order to store more lipids and/or to enlarge the contact area with other organelles (Ibayashi et al., 2021). Moreover, substantial metabolic switches after EGA also affect the lipid profile, lipids utilization and metabolic pathways. The TAG and cholesterol esters are the most dynamic (with the largest quantitative changes) lipid subclasses comparing the early development stages (2-cell and 8-cell embryos) to the blastocyst stage of bovine embryos (de Lima et al., 2018; Milazzotto et al., 2022). Furthermore, LD number changes during the sequential stages of embryogenesis in cattle, however, not all stages have been described (Sudano et al., 2016). It has been also suggested that lipid droplets distribution may reflect the metabolic state of an oocyte or embryo since LD dispersion is linked with a more efficient β-oxidation process while LD fusion with increased glycolysis (Bradley and Swann, 2019).

Lipid metabolism of oocytes and embryos is affected by many factors, e.g., lipid content noticeably varies among species. The contradictory lipid content values are observed in porcine oocytes (156 ng of total free fatty acids content) and murine (4 ng), while bovine takes intermediate values—58 ng (Bradley and Swann, 2019). That disproportion is also observed in the embryos. Porcine embryos, highly rich in lipids, can develop relying on lipids as the only energy source while mouse embryos lipid metabolism and lipid content tend to be low since they utilize pyruvate as the main source of energy (Bradley and Swann, 2019). For bovine embryos, the high intracellular lipid level can lead to metabolic defects and directly to compromised development (Ferguson and Leese, 1999; Shi and Sirard, 2022). It is suggested that bovine and porcine embryos may require different culture conditions to support their energy demands in the *in vitro* system. Therefore expanding knowledge about lipid metabolism in subsequent steps of preimplantation development of both species is expected.

Another factor, that may affect lipid metabolism, is embryo genome origin. Mammalian oocytes can be parthenogenetically activated *in vitro*, without the involvement of a male gamete. There are several mechanisms of parthenogenetic activation (PA) known in nature and applied to laboratory experiments, however, the one that allows analyzing the impact of paternal genome deficiency on embryo development considers the resumption of meiosis by chemical activation without second polar body extrusion. It results in the formation of a diploid zygote composed solely of maternal DNA. The mammalian PA embryos can develop to various stages of development depending on the species but never to term. Porcine parthenogenetic embryos may develop up to day 29 (Kure-Bayashi et al., 2000). A significant impact of paternal genome contribution in embryo development has been documented by 25 differentially expressed imprinted genes involved in, e.g., cell proliferation, growth and differentiation, RNA processing and apoptosis between IVF and PA porcine embryos (collected at day 28 or 30) (Bischoff et al., 2009). This is due to the fact that selected genes are subjected to parental imprinting. As a result, the paternal or maternal origin of an allele affects its transcriptional activity. Additionally, there is a difference in the most active stage of transcription—morula stage for PA and blastocyst stage for IVF porcine embryos revealed by the largest number of differentially expressed genes during these stages. The PA embryos also showed enrichment in apoptosis processes (Li et al., 2021). No data on the lipid metabolism in PA vs. IVF embryos have been previously reported, which seems like a very interesting issue. It would indicate whether genome imprinting disruptions affect, e.g., lipid storage or catabolism, which might be negative for embryo development.

The objective of the present work was to perform complex characterization of lipid droplets during subsequent developmental stages of porcine and bovine embryos. Moreover, two main factors with potential effects on LD have been studied: species-specificity (bovine vs. porcine) and embryo genome origin (IVF vs. PA).

## 2 Materials and methods

Unless otherwise stated, all reagents were supplied by Merck Group.

### 2.1 Collection of cumulus-oocyte complexes

The oocyte-cumulus complexes (COCs) were obtained from bovine and porcine ovarian follicles (3–5 mm diameter). Immature COCs were collected by aspiration with a needle and syringe and analyzed morphologically in HEPES medium. COCs with at least 3-4 layers of cumulus cells and without signs of degradation cytoplasm were selected for the experiment.

### 2.2 *In vitro* maturation

*In vitro* maturation (IVM) was performed in four-well (Nunc) plates in 500 µL of maturation medium under conditions: 5% CO_2_ in the atmosphere, 38.5°C and maximum humidity (for porcine) and at 39°C (for bovine), 5% CO_2_ at maximum humidity.

Bovine COCs were matured in the following medium: TCM199+Glutamax (Gibco, Thermo Fisher Scientific, MA, United States) supplemented with 6 mg/mL fafBSA (fatty acid free bovine serum albumin), 0.25 mM Na pyruvate, 1x concentrated penicillin-streptomycin solution, 2 μg/mL FSH and 1 μg/mL β-estradiol for 24 h.

For porcine IVM, the first 24 h included NCSU-23 medium (North Carolina State University Medium-23) supplemented with hormones: 10 U PMSG (pregnant mare serum gonadotropin, Chorulon, MSD Animal Health, NL, United States), 10 U hCG (Folligon, MSD Animal Health, NL, United States) and 10% of follicular fluid. Next COCs were transferred to a fresh medium without hormones for 20 h (Pawlak et al., 2018). After maturation, COCs were either denuded and parthenogenetically activated (bovine and porcine) or COCs with cumulus cells included were *in vitro* fertilized (bovine).

### 2.3 *In vitro* fertilization/parthenogenetic activation, embryo culture

For bovine *in vitro* fertilization (IVF), good quality semen from two bulls was purchased from the commercial artificial insemination station. The motile fraction of the sperm was selected with BoviPureTM System according to the producer protocol (Nidacon, Mölndal, Sweden). Shortly, semen was thawed and centrifuged in two layers of 80% and 40% BoviPureTM solution at 300 g × 15 min, then the precipitate was washed in BoviWashTM solution. Final semen concentration was counted under the microscope (Nikon YS2-T) in Burker Chamber (BRANDT) and gamete coincubation was carried out with a semen concentration of 1 × 10^6^/ml for 20 h. IVF medium was supplemented with PHE (penicillin, hypotaurine, epinephrine).

Parthenogenetic activation of denuded bovine and porcine oocytes was carried by incubation in 5 µM Ionomycin in TALP-medium for 5 min followed by 4 h incubation in 2 mM 6-DMAP (6-(Dimethyloamino)purine) in final embryo culture medium supplemented with BSA.

SOF+fafBSA (for bovine; Warzych et al., 2020) and NCSU23/NCSU23+FBS (for porcine; Pawlak et al., 2020); media were used as embryo culture media up to the expanded blastocyst stage. On day 5 post-insemination/post-activation, half of the drop volume was replaced with fresh medium.

Embryos in subsequent stages of preimplantation development were collected at specific time points listed in Table 1. Three experimental groups were designed: b.IVF (bovine embryos after *in vitro* fertilization), b.PA (bovine embryos after parthenogenetic activation) and p.PA (porcine embryos after parthenogenetic activation).

**TABLE 1:** Porcine and bovine embryos in subsequent development stages with exact collection times.

Embryonic stage	Time after gamete coincubation (*in vitro* fertilization) or after parthenogenetic activation [h]	
	Pig (h)	Cattle (h)
Zygote	4	20
2-cell embryo	24	30
4-cell embryo	54	48
8–16-cell embryo	92	80
Morula	104	120
Early blastocyst	140	168
Blastocyst	150	192

### 2.4 Lipid droplet staining, microscope analysis and image analysis

Embryos were fixed in 4% PFA (paraformaldehyde) for 30 min in a four-well (Nunc) plate. PFA was removed by 3x washing in PBS+0.2% PVP (5 min) and stored at 4°C. Fixed embryos were permeabilized with 0.2% Triton X-100 solution for 20 min at room temperature and washed 3x in PBS+0.2% PVP (5 min). For lipid droplets staining, 20 μg/mL BODIPY 493/503 dye (Thermo Fisher Scientific, MA, United States) was used at room temperature for 1 h. Additionally, chromatin was visualized by staining with 0.5 μg/mL Vectashield DAPI (4′,6-diamidino-2-phenylindole (DAPI; Vector Laboratories, Burlingame, CA, United States) and cytoskeleton by 1x Phalloidine for more accurate cell contours visualization (iFluor 555 Reagent, Abcam, Cambridge, United Kingdom). Embryos (from zygotes to early blastocysts) were mounted on glass slides with single concave (Comex, PL). For expanded blastocyst, µ-Slide, 18 Well-Flat ibiTreat plates (ibidi, GmbH, Germany) with polylysine instead of glass slides were used. Embryos were analyzed using a confocal microscope Zeiss LSM 880 using 488 nm filter with bandpass 500–550 nm for BODIPY 493/503 (laser Argon2) and 420–480 nm for DAPI (laser Diode 405) and 567–650 nm for Phaloidine with LD LCl Plan Apochromat 40x/1.2 Imm Korr DIC 27 objective (Zeiss, Germany). Each embryo was photographed in 5 μm intervals, from the top slice (with the first capture of LD) to the equatorial section. Extended blastocysts were situated on the slide in a certain way to allow confocal visualization of both inner cell mass and trophectoderm. ICM and TE cells were distinguished based on cell shape. For the entire experiment, the settings for bovine (IVF and PA) and porcine embryos remained unchanged. Obtained images were analyzed in terms of lipid parameters [lipid content, lipid droplets number, lipids droplets size and area occupied by lipid droplets (%)] with the ImageJ Fiji software version v1.53c (NIH, Bethesda, MD, United States). Lipid content was assessed with the following formula: integrated density value—(total embryo area value x background fluorescence value). The remaining parameters (LD number, size and area) were measured using the “analyze particle” command, which recognized and read the fluorescence signals that stood out above the photo background. The area occupied by lipid droplets (%) was automatically calculated on the basis of fluorescence to non-fluorescence signal ratio. The additional use of the “watershed” command enabled to eliminate possible LD clusters generated by the Fiji software. For all analyzed parameters, the value of each slice was calculated separately and the mean value per slice was the final result of the calculation. It avoided high variability due to the size variation among embryo stages. In order to analyze the lipid content within the inner cell mass in relation to the entire expanding blastocyst, additional measurements (according to the protocol) were made for the area occupied by the ICM.

### 2.5 Statistical analysis

The analysis of the distribution of the collected embryos was carried out by the Shapiro-Wilk test and statistical significance comparison by Wilcoxon test and the Kruskal-Wallis test in statistical package R (https://cran.r-project.org/). The comparison between ICM of expanded blastocysts was performed with a chi-square test.

## 3 Results

Embryos of both species and originating from either IVF or PA systems at each stage of development were collected from 3 to 5 *in vitro* production replicates. More than one developmental stage have been collected from a single experimental replicate. The cleavage and blastocysts rates were as follows: b.IVF—80% and 20%, b.PA—79% and 17%, p.PA—70% and 26%.

### 3.1 Bovine IVF embryos lipid characterization

**FIGURE 1:**
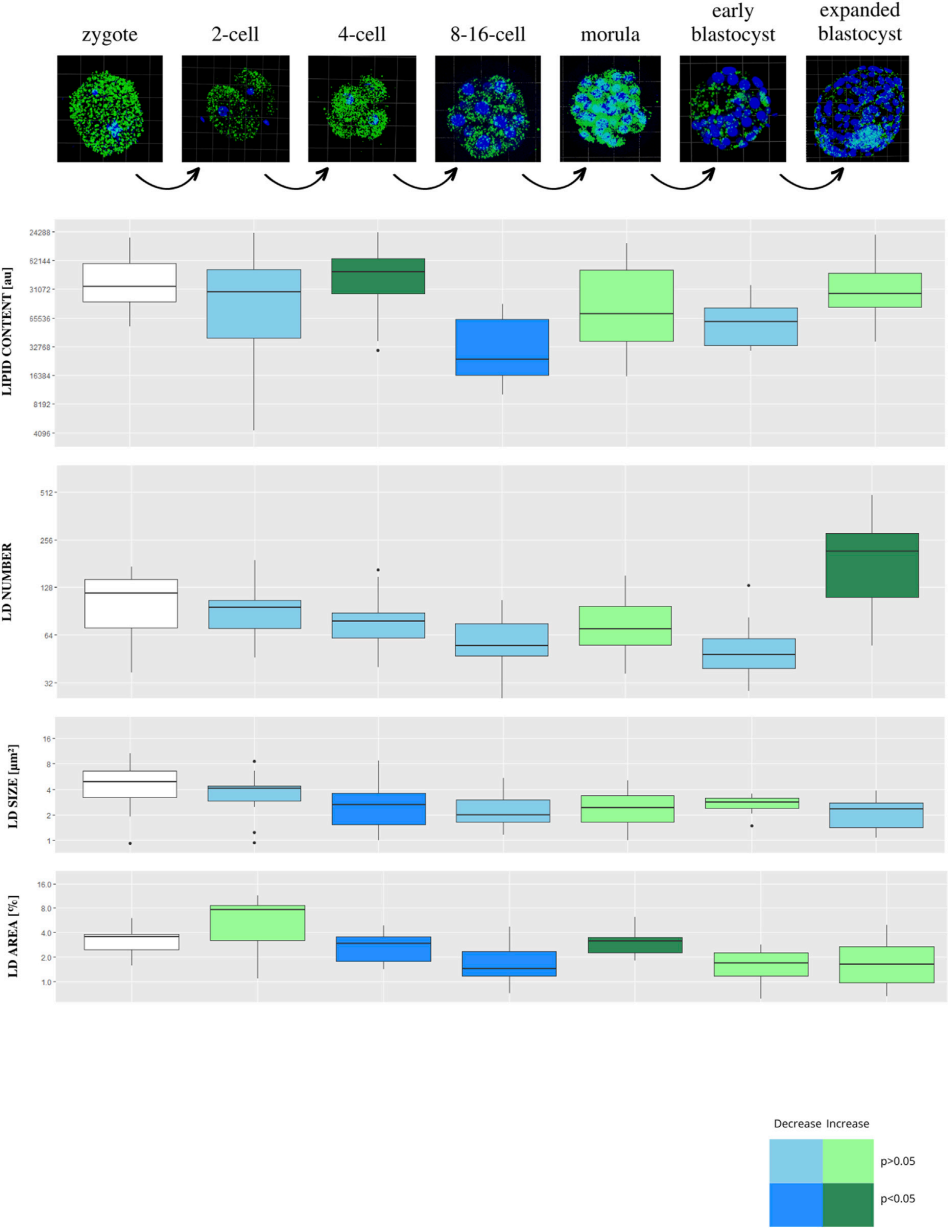
The diagram presents subsequent developmental stages of bovine IVF embryos, with lipid droplets parameters included. Each stage of development is compared to the one step before (2-cell vs. zygote, 4-cell vs. 2-cell, 8/16-cell vs. 4-cell, morula vs. 8/16-cell, early blastocyst vs. morula, expanded blastocyst vs. early blastocyst). The dark blue color of a box indicates significant drop of the parameter, whereas dark green—significant increase of the parameter. The light colors indicate up- or down- changes but not significant. The subsequent rows of the figure present: confocal 3D visualization of embryos, green dye—lipid droplets, blue dye–nuclei; Lipid content parameter mean value per slice ± SD; LD number parameter mean value per slice ± SD; LD size parameter mean value per slice ± SD; percentage LD area parameter mean value per slice ± SD. The logarithmic transformation of the values on the Y-axis has been applied.

Lipid parameters (lipid content, lipid droplets number, lipid droplets size and percentage area occupied by lipid droplets within the embryo) were analyzed in 131 individual bovine IVF embryos (b.IVF) from 7 developmental stages (zygote, n = 19; 2-cell, n = 18; 4-cell, n = 20; 8–16-cell, n = 18; morula, n = 22; early blastocyst = 20; expanded blastocyst, n = 15). Confocal visualization and computer analysis revealed numerous significant alterations between subsequent development stages. A comparison between each stage of embryo development vs. the previous stage is presented in Figure 1 (2-cell vs. zygote, 4-cell vs. 2-cell, 8–16-cell vs. 4-cell, morula vs. 8–16-cell, early blastocyst vs. morula, expanded blastocyst vs. early blastocyst). The summarized total statistical results are shown in the supplementary data (Supplementary Table S1).

There is a decrease in lipid content and the area occupied by lipid droplets parameters between early developmental stages, reaching the lowest point at 8–16-cell stage. The highest value of lipid content is observed at the 4-cell stage. A gradual decrease is observed in lipid droplets number from zygote up to 8–16-cell stage, moreover, a significant increase of this parameter is observed at the expanded blastocyst stage. Lipid droplets size scores the highest value in zygotes, while after a significant decrease at the 4-cell stage, it remains constant in subsequent stages.

### 3.2 Bovine PA embryos lipid characterization

**FIGURE 2:**
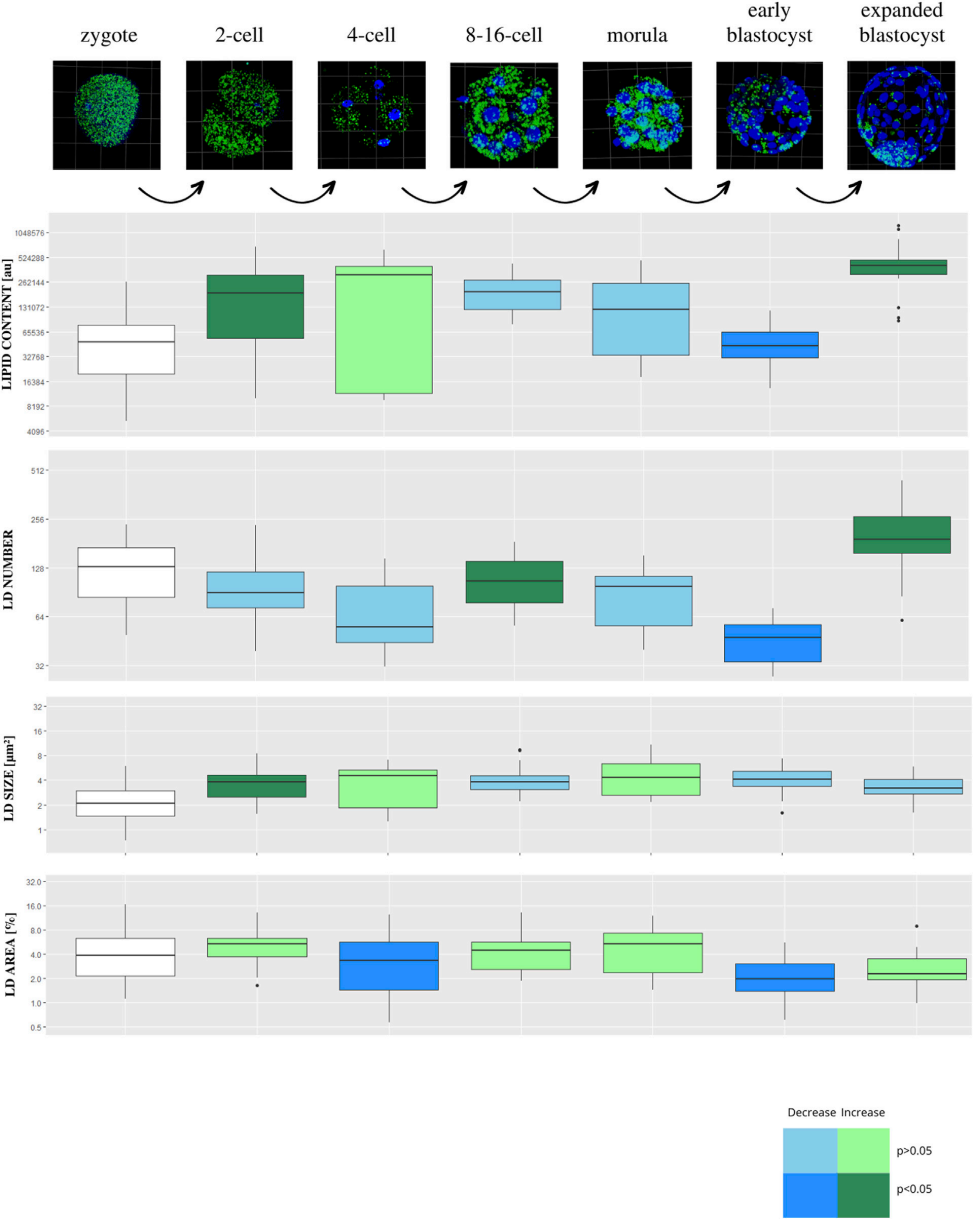
The diagram presents subsequent developmental stages of bovine PA embryo development, with lipid droplets parameters included. Each stage of development is compared to the one step before (2-cell vs. zygote, 4-cell vs. 2-cell, 8/16-cell vs. 4-cell, morula vs. 8/16-cell, early blastocyst vs. morula, expanded blastocyst vs. early blastocyst). The dark blue color of a box indicates significant drop of the parameter, whereas dark green—significant increase of the parameter. The light colors indicate up- or down- changes but not significant. The subsequent rows of the figure present: confocal 3D visualization of embryos, green dye—lipid droplets, blue dye–nuclei; Lipid content parameter mean value per slice ± SD; LD number parameter mean value per slice ± SD; LD size parameter mean value per slice ± SD; percentage LD area parameter mean value per slice ± SD. The logarithmic transformation of the values on the Y-axis has been applied.

Lipid parameters (lipid content, lipid droplets number, lipid droplets size and percentage area occupied by lipid droplets) were analyzed in 123 individual bovine parthenogenetic embryos (b.PA) from 7 development stages (zygote, n = 19; 2-cell, n = 20; 4-cell, n = 14; 8–16-cell, n = 18; morula, n = 22; early blastocyst = 15; expanded blastocyst, n = 15). Confocal visualization and computer analysis revealed numerous significant alterations between subsequent development stages. A comparison between each stage of embryo development vs. the previous stage is presented in Figure 2 (2-cell vs. zygote, 4-cell vs. 2-cell, 8–16-cell vs. 4-cell, morula vs. 8–16-cell, early blastocyst vs. morula, expanded blastocyst vs. early blastocyst). The summarized total statistical results are shown in the supplementary data (Supplementary Table S2).

We found a significant drop in values for lipid content and LD number parameters at the early blastocyst stage followed by a sudden increase in the expanded blastocyst stage. The analysis also shows that the lipid droplets size parameter was the lowest in the zygote stage, then it increased significantly in the 2-cell stage, staying stable during further stages of development.

### 3.3 Porcine PA embryos lipid characterization

**FIGURE 3:**
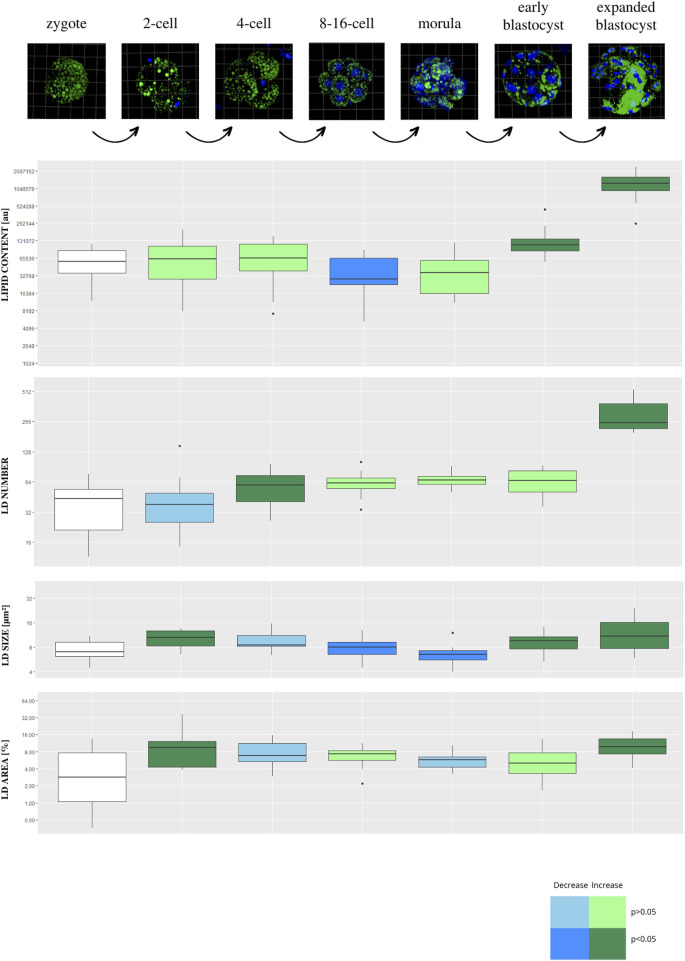
The diagram presents subsequent developmental stages of porcine PA embryo development, with lipid droplets parameters included. Each stage of development is compared to the one step before (2-cell vs. zygote, 4-cell vs. 2-cell, 8/16-cell vs. 4-cell, morula vs. 8/16-cell, early blastocyst vs. morula, expanded blastocyst vs. early blastocyst). The dark blue color of a box indicates significant drop of the parameter, whereas dark green—significant increase of the parameter. The light colors indicate up- or down- changes but not significant. The subsequent rows of the figure present: confocal 3D visualization of embryos, green dye—lipid droplets, blue dye–nuclei; Lipid content parameter mean value per slice ± SD; LD number parameter mean value per slice ± SD; LD size parameter mean value per slice ± SD; percentage LD area parameter mean value per slice ± SD. The logarithmic transformation of the values on the Y-axis has been applied.

Lipid parameters (lipid content, lipid droplets number, lipid droplets size and percentage area occupied by lipid droplets) were analyzed in 130 individual porcine parthenogenetic embryos (p.PA) from 7 development stages (zygote, n = 21; 2-cell, n = 21; 4-cell, n = 19; 8–16-cell, n = 21; morula, n = 17; early blastocyst = 20; expanded blastocyst, n = 11). Confocal visualization and computer analysis revealed numerous significant alterations between subsequent development stages. A comparison between each stage of embryo development vs. the previous stage is presented in Figure 3 (2-cell vs. zygote, 4-cell vs. 2-cell, 8–16-cell vs. 4-cell, morula vs. 8–16-cell, early blastocyst vs. morula, expanded blastocyst vs. early blastocyst). The summarized total statistical results are shown in the supplementary data (Supplementary Table S3).

The expanded blastocyst stage was characterized by the highest values in all analyzed parameters. The lipid content was decreasing at 8–16 and morula stage and peak at the highest values in the expanded blastocyst. The gradual increase of LD number was observed from the zygote up to the expanded blastocyst stage which achieved the highest values.

### 3.4 Bovine IVF vs. bovine PA

**FIGURE 4:**
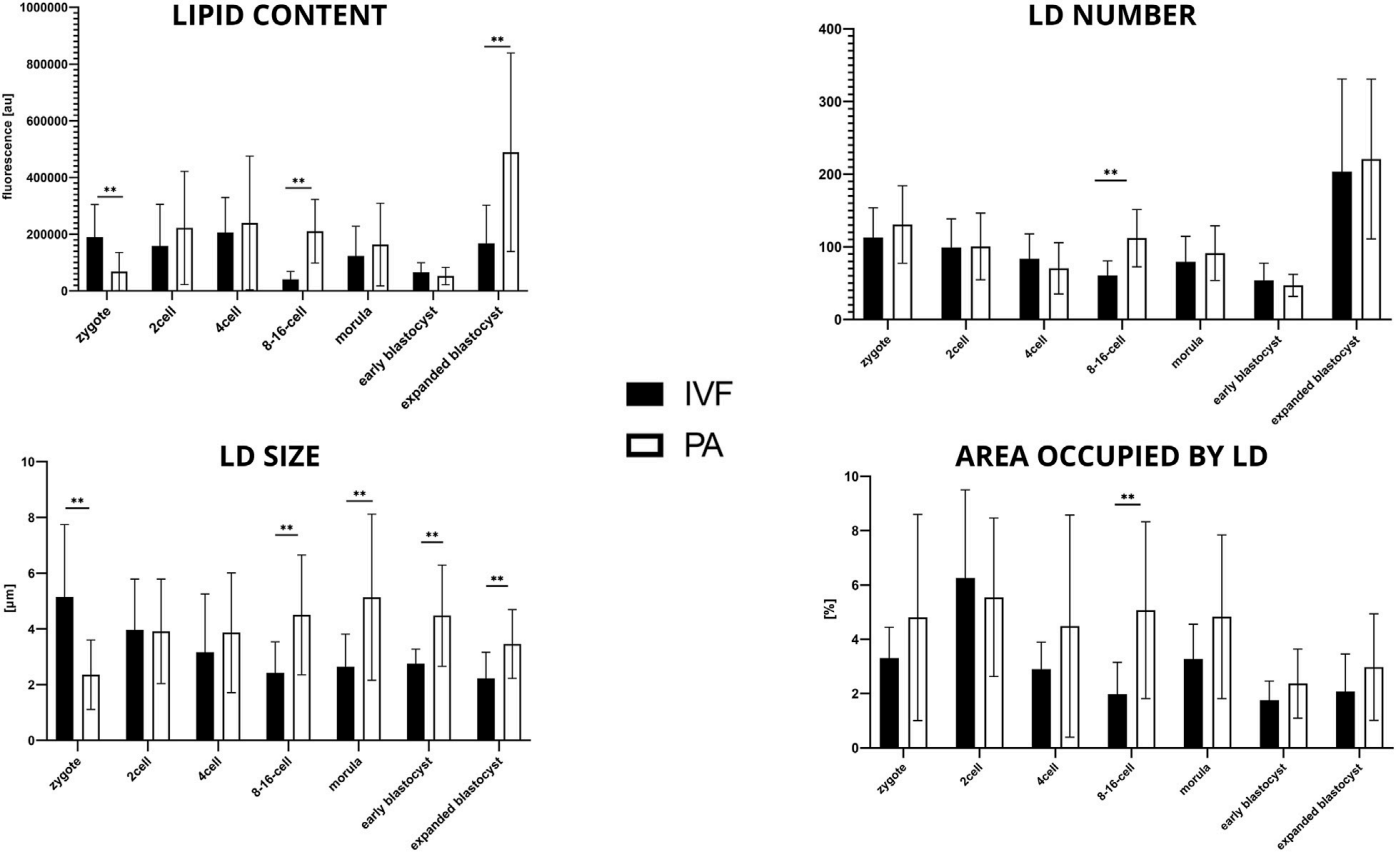
The results of lipid parameters comparison (mean ± SD) between bovine IVF and bovine PA groups in subsequent embryo development stages.

The first noticeable difference between the two groups is the significantly lower level of lipid content and LD size in b.PA zygote stage (*p* < 0.01), while the LD number and the average area occupied by LD remain the same. Next, significantly lower values (*p* < 0.01) in b.IVF 8–16-cell embryos were observed in all analyzed parameters. The size of LDs increased from 8–16-cell stage embryos up to the blastocyst stage. The higher lipid content in b.PA expanded blastocyst (*p* < 0.01) corresponds with higher LD sizes (*p* < 0.05) when compared to b. IVF (Figure 4).

### 3.5 Bovine PA vs. porcine PA

**FIGURE 5:**
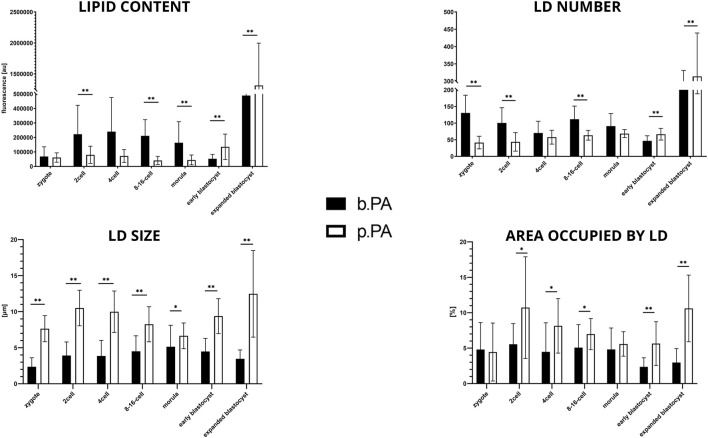
The results of lipid parameters comparison (mean ± SD) between bovine PA and porcine PA groups in subsequent embryo development.

Considering lipid content analysis at the zygote stage, the lipid level is similar between b.PA and p.PA. Embryos at the 2-cell and 8–16-cell stages as well as the morulas had significantly (*p* < 0.01) more lipids in b.PA group. The relation was opposite in the early and expanded blastocysts, where lipid content was significantly higher in the p.PA embryos (*p* < 0.01). Moreover, in b.PA group more lipid droplets were observed in the zygote, 2-cell and 8–16-cell stages (*p* < 0.01) and likewise, it resulted in a significantly higher number of LD in p.PA blastocysts (*p* < 0.01). LD size was higher in all development stages of p.PA embryos. Moreover, except for the zygote and morula, the area occupied by LD was larger in the p.PA group (Figure 5).

### 3.6 ICM vs. entire blastocyst comparison

The measurements within expanded blastocysts regarding lipid content in the inner cell mass in relation to the total embryo have been performed. Results show that lipid content within the ICM was 51% ± 22%, 52% ± 16% and 42% ± 23% for b.IVF, b.PA, and p.PA respectively of total embryo lipid capacity (summarized in Table 2). There were no significant differences when the ICM parameters were compared between b.PA vs. p.PA and b.IVF vs. b.PA groups. Although there are differences between general lipid content in blastocyst (shown in comparisons described in paragraphs above), the ICM results indicate that in all groups lipids are distributed with the same pattern—nearly evenly between ICM and TE cell lines.

**TABLE 2:** The results of expanded blastocyst analysis with lipid content [%] in ICM in relation to the entire blastocyst; green dye–lipid droplets, blue dye–nuclei; ICM–inner cell mass, BL–blastocyst.

	b.IVF	b.PA	p.PA
Expanded blastocyst visualization	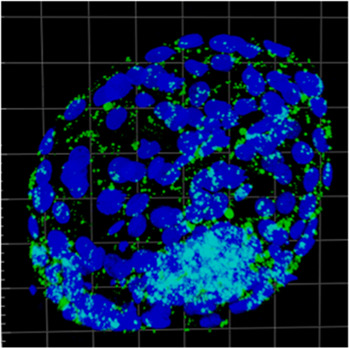	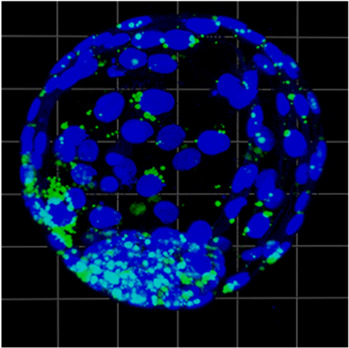	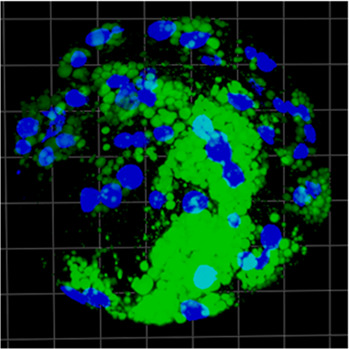
Lipid content in ICM [au]	86,844 ± 67,481	215,483 ± 170,366	476,046 ± 321,139
Lipid content in ICM per entire blastocyst [%]	51 ± 22	52 ± 16	42 ± 23

## 4 Discussion

Lipids are essential components of the cells since they participate in cellular membranes formation and many key processes such as cell division, differentiation or signaling cascades. Research has shown that lipid metabolism and lipid droplet dynamics are regulated by a variety of factors. The alterations in lipid metabolism can result in oocyte developmental defects and reduced fertility as well as impaired embryo development (Shi and Sirard, 2022). Maintaining adequate levels of LDs is important both for oocytes and early embryos, even in mammalian species with low LDs number in the oocytes (Aizawa et al., 2019). A possible imbalance in lipid content can negatively affect not only lipid metabolism itself but also cellular homeostasis which can lead to reduced embryo quality (Ibayashi et al., 2021). Therefore, we decided to characterize lipid droplets parameters within two mainly used large animal models–cattle and pigs, as well as, evaluate the impact of embryo genome origin (IVF vs. PA).

In the presented study, we evaluated several lipid parameters (lipid content, lipid droplet number, lipid droplet size and area occupied by lipid droplets) and compared them among subsequent development stages of bovine *in vitro* preimplantation embryos. Among others, we noticed the lowest values of all analyzed parameters at the 8–16-cells stage bovine embryos, which coincides with the time of bovine embryo genome activation. It has been previously suggested that EGA is in high energy demand due to large-scale gene transcription (Zhai et al., 2022). This hypothesis is supported by the transcriptomic results of Milazzotto et al. (2022), who showed increased expression of genes involved in TAGs catabolism at the 8-cell stage when compared to the earlier stage. Therefore, our lipid content observations might suggest that during bovine EGA strong TAGs catabolism takes place, which results in a decrease in lipid content, to produce a high amount of energy. Sudano et al. (2016) did not observe a decrease in the lipid content at the 8–16-cell stage (droplets stained with Neil Red), however, their analysis was performed with a simple fluorescence microscope and each embryo was analyzed only in the equatorial plane (Sudano et al., 2016). The present experiment was performed with confocal microscopy with a higher precision due to 3D embryo reconstruction. Therefore, to our knowledge, we are the first to show that along with high energy requirements and high glucose consumption, the bovine embryo also consumes lipids more intensively during EGA.

The second interesting outcome from bovine IVF embryos analysis was the significant increase of lipid droplets number in the expanded blastocyst stage, which suggests the demand for energy accumulation at this stage of development. It agrees with the results of Miazzotto et al. (2022), who observed the upregulation of genes involved both in carbohydrate and lipid metabolism in blastocysts, including TGAs biosynthesis in bovine blastocysts. Moreover, glycolysis genes were not affected in blastocysts (Milazzotto et al., 2022). It is rather surprising since previously it has been suggested that energy production from carbohydrates is preferable at this stage of development (Leese, 2012). However, we suggest that lipids are still of high importance for the proper bovine embryo development at the blastocyst stage, despite the general switch into energy production mainly from glucose source (Leese, 2012). It might be related to the fact, that at the blastocyst stage intensive mitotic divisions take place, which requires the formation of membranes for new cells, for which lipids are required. Also, ICM/TE and blastocoel formation as well as further implantation is energy demanding (Ibayashi et al., 2021) hence embryos store LD intensively for further proper development. This hypothesis has been recently supported by the studies of Mau et al. (2022), who revealed accumulation of LDs around the time of implantation of mice embryos is necessary to support peri-implantation development.

Oocytes can be successfully activated without male gamete by inducing them to undergo multiple cleavage divisions, thus initiating embryonic development in the process of parthenogenesis. The developmental arrest of the parthenotes (parthenogenetically activated embryos) is due to changes in their genomic imprinting (Kirioukhova et al., 2018). In the present experiment, in bovine parthenogenetic embryos, we observe an increase of lipid content from the zygote to the 2-cell stage, followed by a decrease at the early blastocyst stage and a further increase in the expanded blastocyst. Therefore, no significant changes around the EGA process are observed and strong lipid catabolism is noticed only when blastocysts are formed. Also, the LD number significantly decreases at the early blastocyst stage and further increases at the expanded blastocyst stage, suggesting a possible energy demand at this stage of development.

To reveal the impact of parthenogenetic activation on lipid droplets parameters in preimplantation embryos, bovine PA embryos were compared with bovine IVF counterparts. There were three main observations: 1) b.PA zygotes have significantly fewer lipids (*p* < 0.01) than b.IVF; 2) b.PA embryos at the 8–16-cell stage have significantly higher values in all analyzed parameters than b.IVF (*p* < 0.01), and 3) b.PA expanding blastocysts contain significantly more lipids with a bigger size of lipid droplets when compared to b.IVF. According to the literature, both first cleavage and DNA synthesis occur earlier in bovine PA embryos than in IVF embryos (summarized by Gómez et al., 2009). Moreover, it was shown that parthenogenetic activation disturbs the total protein profiles of porcine zygotes (Gupta et al., 2009), which suggests possible substantial abnormalities in the basic metabolism. During standard fertilization, sperm induces degradation of MPF activity (a protein kinase that inhibits meiosis division) via repeated rises of calcium ion levels (Miao and Williams, 2012). In the PA protocols, chemical or physical agents induce this mechanism. We suggest that zygotes during parthenogenetic activation might reveal higher energy consumption due to the artificially induced processes. Surprisingly, the opposite situation is observed at the 8–16-cell stage, when significantly lower values are found for all lipid parameters in the IVF group compared to PA. Since this is a moment of bovine embryo genome activation, two explanations are possible. One is that b.IVF embryos, containing both maternal and paternal genetic material and undergoing demethylations of both, require more energy during this transition than parthenotes consisting of a duplicate set of an epigenetically imprinted maternal genome. Another hypothesis is that b.PA embryos are impaired with regard to the expression of selected genes controlling lipid metabolism (especially the ones related to lipolysis, which is a process of TAGs hydrolysis) because of only maternal methylation pattern. Due to the lack of other publications focused on this subject, it is highly interesting for further studies. In the present experiment, there is also observed significantly higher accumulation of lipids in b.PA blastocysts when compared to b.IVF counterparts. Studies on other species showed that parthenogenetic activation does not affect glucose metabolism in goat blastocysts (Ongeri and Krisher, 2001), whereas it dysregulates lipid metabolism (Pu et al., 2020). We also observed higher lipid content in PA blastocysts. All those observations might be due to the dysregulation of lipolysis processes in parthenogenetic embryos, which is however only a hypothesis.

The next goal of the present experiment is the analysis of lipid droplets parameters in the porcine parthenogenetic preimplantation embryos. We observe high variability between stages in every analyzed parameter. The most interesting is the significant decrease in lipid content at the 8–16-cell stage. In *in vivo* porcine embryos the EGA occurs during the 4-cell stage, yet the literature data points that under *in vitro* conditions, this transition is postponed to the next stage (Piliszek and Madeja, 2018). Therefore LD utilization at the 8–16 cell stage may be related to the intensive energy demand due to the embryo transcription onset. On the contrary, Sturmey and Leese, 2003(2003), as well as Romek et al. (2011) showed that there were no significant changes in triglyceride content within the developmental stages of the pig embryo. These authors suggested that this may be due to variation within the number of samples used in the experiments. Hence, we propose that to some extent embryo genome activation may influence the observed decrease of parameters at the subsequent 8–16-cell stage in porcine PA embryos. It is also interesting to note that the highest parameter values in porcine embryos are observed in expanded blastocysts. This suggests a high demand for energy storage at this stage of porcine embryo development, similar to the bovine blastocysts. During blastocyst development specific cell differentiation occurs, ICM and TE cells are observed and the embryo is prepared for the process of implantation. As implantation is a demanding process (Downs et al., 2009; Wang et al., 2013), lipid storage appears to be energetically advantageous for the blastocyst. However, since lipid storage capacity varies between species, the potentially optimal amount of lipids required for proper development must also remain species-specific (Bradley and Swann, 2019).

In the present experiment, we also aimed to compare bovine and porcine embryos throughout the preimplantation development stages. According to the b.PA and p.PA embryos evaluation, the first noted data was the lack of differences in the lipid content between zygotes of both species. This is rather surprising since porcine oocytes have much higher lipid content than other farm animal species. It has been previously shown that LD number and morphology differ among porcine *in vivo*, *in vitro* and parthenogenetic embryos (Kikuchi et al., 2002). Therefore, it might be suggested that porcine zygotes utilize lipids more intensively than bovine ones, which results in equal lipid content at this stage of development. Next, we observe lower lipid content in porcine PA embryos when compared to bovine ones between 2-cell and morula stages. However, at the same time of development, porcine embryos are characterized by higher LD size and area occupied by LD. Therefore, in our opinion, this part of the results indicates variable individual characteristics of lipid droplets between these two species, the origin of which is difficult to explain based on the present experiment. The opposite results are shown in the early and expanded blastocysts, where higher lipid parameters are observed in porcine embryos, which is in agreement with the results of Kajdasz et al. (2020). Furthermore, their RNA-seq results showed the upregulation of genes related to glucose metabolism as a preferable source of energy for porcine PA blastocysts unlike bovine blastocysts (Kajdasz et al., 2020). It is therefore suggested that porcine PA blastocysts, similarly to bovine, switch partially to glucose metabolism, however they still strongly store lipids without excessive utilization. The putative role of this process is however unknown. Another result that draw our attention was the size and the number of lipid droplets. Porcine LD tend to be bigger but fewer compared to bovines. We suspect that this is a species-specific trait, since similar data have been previously published on intracellular LD of porcine (Lee et al., 2019) and bovine (Lipinska et al., 2021) oocytes.

During the preimplantation embryo development, the very first differentiation events occur already around the morula stage and continue during the blastocyst stage, when the inner cell mass and trophectoderm are formed (Piliszek and Madeja, 2018). Since both cell lines have diverse functions, their metabolic demand also differs. The TE cells have higher pyruvate and lower glucose consumption, while in ICM a contradictory effect is observed (Gopichandran and Leese, 2003). Being aware of the differences in carbohydrate metabolism, we decided to analyze the lipid content within ICM in relation to the whole blastocyst. In all analyzed cases (b.IVF, b.PA, p.PA) we observed the same pattern—lipids were allocated equally between ICM and TE. It is however known that the total area of the cells is not evenly divided between these two lines, because ICM covers only a third part of the blastocyst area (Gopichandran and Leese, 2003). We suggest that ICM cells store lipids more intensely due to their pluripotency and their further properties (Diamante and Martello, 2022). Moreover, lipids that take part in signaling cascades (e.g., arachidonic acid) are of the highest importance for pluripotent cells development (Yanes et al., 2010). The lack of differences between analyzed groups (b.IVF vs. b.PA and b.PA vs. p.PA) can be explained by the same molecular mechanism (CDX2 and OCT-4 expression) that is involved and essential in the ICM/TE differentiation in both bovine and porcine embryos (Emura et al., 2016; Sakurai et al., 2016).

## 5 Conclusion

In the presented study, we analyzed porcine and bovine subsequent preimplantation embryo stages in terms of species-specificity and genome-origin possible impact on lipid metabolism and we have shown multiple differences. Lipid parameters in the IVF vs. PA bovine embryos differ at the most crucial moments of embryonic development (zygote, 8–16-cell, blastocyst), indicating possible dysregulations of lipid metabolism in PA embryos. When bovine vs. porcine species are compared, we observe higher lipid content around the EGA stage and lower lipid content at the blastocyst stage for bovine embryos, which indicates different demands for energy depending on the species. We conclude that lipid droplets parameters significantly differ among developmental stages and between the studied species. Data suggest, that these parameters may be also related to the factors of genome origin. Moreover, the observed pattern of changes during preimplantation development may be applied as one of the indicators of embryo quality and developmental competence under variable *in vitro* culture conditions.

## Data Availability

The original contributions presented in the study are included in the article/Supplementary Material, further inquiries can be directed to the corresponding author.

## Supplementary material

SUPPLEMENTARY TABLE S1 Summarized total results of statistical analysis, where all stages of bovine IVF embryos were compared to each other. There are shown P values of particular comparisons. ns—not significant.

SUPPLEMENTARY TABLE S2 Summarized total results of statistical analysis, where all stages of bovine PA embryos were compared to each other. There are shown P values of particular comparisons. ns—not significant.

SUPPLEMENTARY TABLE S3 Summarized total results of statistical analysis, where all stages of porcine PA embryos were compared to each other. There are shown P values of particular comparisons. ns—not significant.
