# Supplementary material for: Species and embryo genome origin affect lipid droplets in preimplantation embryos
Source: Front Cell Dev Biol. 2023 May 12;11:1187832. doi: 10.3389/fcell.2023.1187832 (PMC10217358; doi:10.3389/fcell.2023.1187832)
Supplement: Supplementary file 3 [file Table3.DOCX]

| **Lipid content** |  | Zygote | 2cell | 4cell | 8-16 cell | Morula | Early blastocyst | Expanded blastocyst |
| --- | --- | --- | --- | --- | --- | --- | --- | --- |
|  | Zygote | x | ns | ns | ns | ns | 0.01 | 0.01 |
|  | 2cell | ns | x | ns | 0.05 | 0.05 | 0.01 | 0.01 |
|  | 4cell | ns | ns | x | 0.05 | 0.05 | 0.01 | 0.01 |
|  | 8-16-cell | ns | 0.05 | 0.05 | x | ns | 0.01 | 0.01 |
|  | Morula | ns | 0.05 | 0.05 | ns | x | 0.01 | 0.01 |
|  | Early blastocyst | 0.01 | 0.01 | 0.01 | 0.01 | 0.01 | x | 0.01 |
|  | Expanded blastocyst | 0.01 | 0.01 | 0.01 | 0.01 | 0.01 | 0.01 | x |
|  |  |  |  |  |  |  |  |  |
| **LD number** |  | Zygote | 2cell | 4cell | 8-16 cell | Morula | Early blastocyst | Expanded blastocyst |
|  | Zygote | x | ns | 0.05 | 0.01 | 0.01 | 0.01 | 0.01 |
|  | 2cell | ns | x | 0.01 | 0.01 | 0.01 | 0.01 | 0.01 |
|  | 4cell | 0.05 | 0.01 | x | ns | ns | ns | 0.01 |
|  | 8-16-cell | 0.01 | 0.01 | ns | x | ns | ns | 0.01 |
|  | Morula | 0.01 | 0.01 | ns | ns | x | ns | 0.01 |
|  | Early blastocyst | 0.01 | 0.01 | ns | ns | ns | x | 0.01 |
|  | Expanded blastocyst | 0.01 | 0.01 | 0.01 | 0.01 | 0.01 | 0.01 | x |
|  |  |  |  |  |  |  |  |  |
| **LD size** |  | Zygote | 2cell | 4cell | 8-16 cell | Morula | Early blastocyst | Expanded blastocyst |
|  | Zygote | x | 0.01 | 0.01 | ns | ns | 0.05 | ns |
|  | 2cell | 0.01 | x | ns | 0.01 | 0.01 | ns | ns |
|  | 4cell | 0.01 | ns | x | 0.05 | 0.01 | ns | ns |
|  | 8-16-cell | ns | 0.01 | 0.05 | x | 0.05 | ns | 0.05 |
|  | Morula | ns | 0.01 | 0.01 | 0.05 | x | 0.01 | ns |
|  | Early blastocyst | 0.05 | ns | ns | ns | 0.01 | x | 0.01 |
|  | Expanded blastocyst | ns | ns | ns | 0.05 | ns | 0.01 | x |
|  |  |  |  |  |  |  |  |  |
| Zygote | 2cell | 4cell | 8-16 cell | Morula | Early blastocyst | Expanded blastocyst |  |  |
| LD area %   Zygote | | x | 0.01 | 0.01 | 0.01 | ns | ns | 0.01 |
|  | 2cell | 0.01 | x | ns | ns | 0.01 | 0.01 | ns |
|  | 4cell | 0.01 | ns | x | ns | 0.05 | 0.05 | ns |
|  | 8-16-cell | 0.01 | ns | ns | x | ns | ns | 0.05 |
|  | Morula | ns | 0.01 | 0.05 | ns | x | ns | 0.01 |
|  | Early blastocyst | ns | 0.01 | 0.05 | ns | ns | x | 0.01 |
|  | Expanded blastocyst | 0.01 | ns | ns | 0.05 | 0.01 | 0.01 | x |
